# A Case of Left Ventricular Masson’s Tumor: Not Every Heart Tumor Is a Myxoma

**DOI:** 10.7759/cureus.40113

**Published:** 2023-06-08

**Authors:** Gabriel Panama, Adolfo Martinez, Majid Yavari, Khalid Saeed Al-Asad, Christopher Hanson

**Affiliations:** 1 Internal Medicine, Michigan State University, East Lansing, USA; 2 Internal Medicine, Michigan State University, Lansing, USA; 3 Department of Cardiology, Sparrow Hospital Thoracic and Cardiovascular Institute, Sparrow Hospital, Lansing, USA

**Keywords:** cardiac mass tumor, left ventricle mass, cardiac magnetic resonance (cmr), intravascular papillary endothelial hyperplasia, masson’s tumor

## Abstract

Masson’s tumor is a benign tumor that usually arises secondary to vascular trauma or thrombi, leading to vascular proliferation. Masson’s tumors are most commonly reported in the head, neck, and extremities. Cases in the heart are exceedingly rare, with most case reports describing the left atrium as the most common location. Even though the tumor is benign, excision is recommended due to the risk of embolization.

This is a case of Masson’s tumor located in the left ventricle. The patient is a 24-year-old female, who presented complaining of palpitations and lightheadedness. Transthoracic echocardiography showed a mobile echodensity in the left ventricle. Cardiac MRI showed characteristics similar to a myxoma. The patient underwent surgical resection and a biopsy showed Masson's tumor. This case report focuses on the histopathological features and imaging findings of Masson's tumor.

## Introduction

Masson’s tumor, also known as Intravascular Papillary Endothelial Hyperplasia (IPEH), is a benign and rare tumor that is more commonly seen in the head, neck, and extremities. It can arise in a pure form or within pre-existing lesions (hemangiomas or thrombi). Pathogenesis involves vascular trauma and stasis stimulating vascular proliferation and endothelial hyperplasia within a blood vessel or a lesion; however, in 70% of the cases, there is no identifiable trigger [1,2]. Histological features include papillary lobules of proliferating endothelial cells in a fibrous stroma within a vascular lumen or within a lesion. Immunohistochemical stains are positive for vascular markers like smooth muscle actin (SMA), factor VIII-related antigens, CD31+, and CD34+, with the latter two being the most sensitive. It is important to differentiate IPEH from malignant neoplasms like angiosarcoma since the latter has the potential to metastasize. Criteria for differentiating from angiosarcoma include: negative CD105+ (a marker present in angiosarcoma), minimal necrosis, and lack of pleomorphic and mitotic activity in cells [2,3].

## Case presentation

History of presentation

The patient is a 24-year-old female who presented initially to her primary care provider complaining of progressive palpitations of a month duration. The episodes lasted approximately 2-3 minutes and occurred unexpectedly with no identifiable trigger. The patient described them as a “heart-racing sensation with a skipping beat” associated with lightheadedness. She denied any chest pain, shortness of breath, or syncope. Her vital signs were within normal limits, and her physical examination was unremarkable. EKG showed sinus rhythm with no abnormal changes. The echocardiogram (echo) revealed a mobile echodensity in the left ventricle (LV) which was concerning for cardiac myxoma. She was instructed to go to the emergency department for further work-up. Her medical history was significant for gastroesophageal reflux disease on Protonix® 40mg daily and migraines on topiramate 75mg once at night.

A repeat echocardiogram with contrast on admission to the hospital demonstrated a normal-sized LV. LV ejection fraction was 55 to 60%. A filling defect was noted in the LV with a size of 1.12 × 0.7 cm which appeared to be close to the mid-inferior wall. All valves were normal. The computed tomography angiography (CTA) coronary angiogram showed normal coronaries with a right dominant system. Thrombophilia work-up was negative.

**Figure 1:**
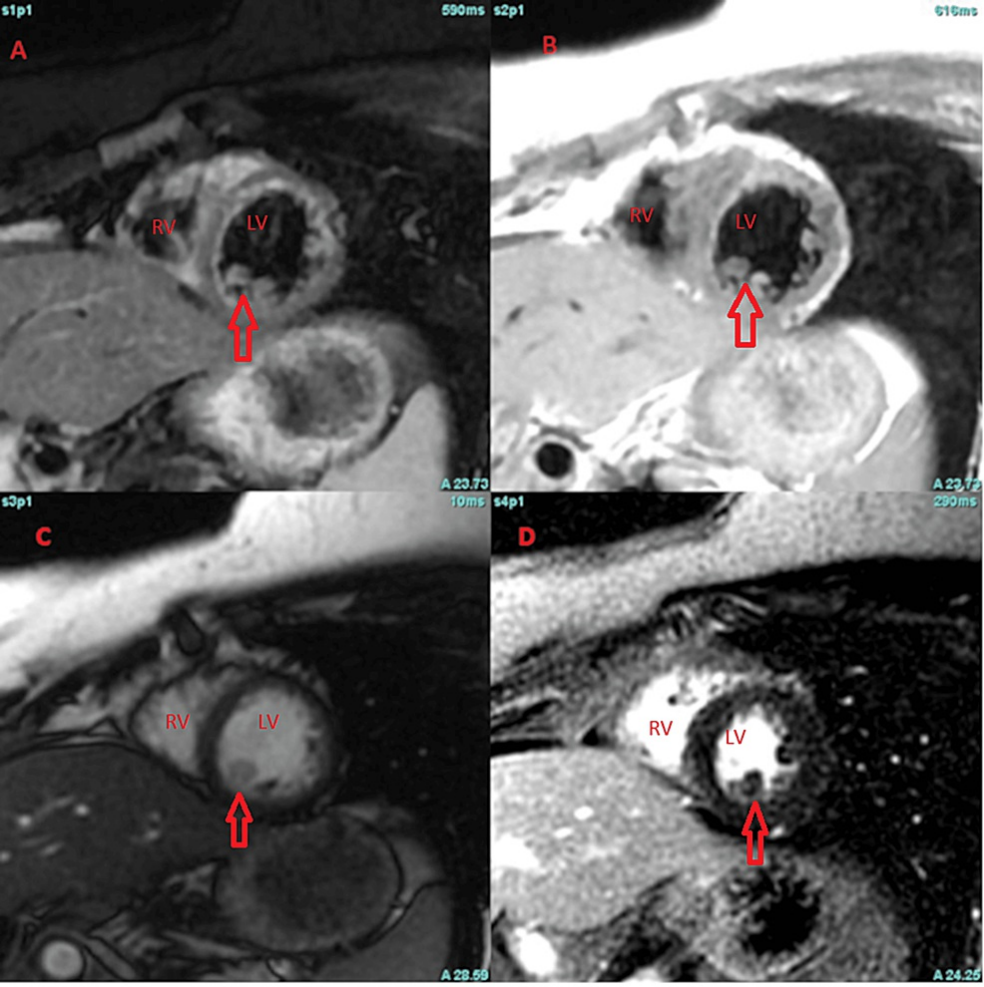
Cardiac MRI imaging A. Fat-suppressed black blood T2 weighted imaging (double Inversion recovery {DIR}, fast spin echo) in a short axis orientation demonstrating the mass to by isointense compared to the myocardium, which is more clearly demonstrated in long axis projections (see Figure 2). B. Black blood T1 weighted imaging (DIR, fast spin echo) in a short axis orientation demonstrating the mass to by isointense compared to the myocardium. C. Steady-state free precession (SSFP) imaging in a short axis orientation demonstrating the mass to by isointense compared to the myocardium. D. Delayed enhancement imaging demonstrating heterogeneous late gadolinium enhancement. LV: Left Ventricle. RV: Right Ventricle

**Figure 2:**
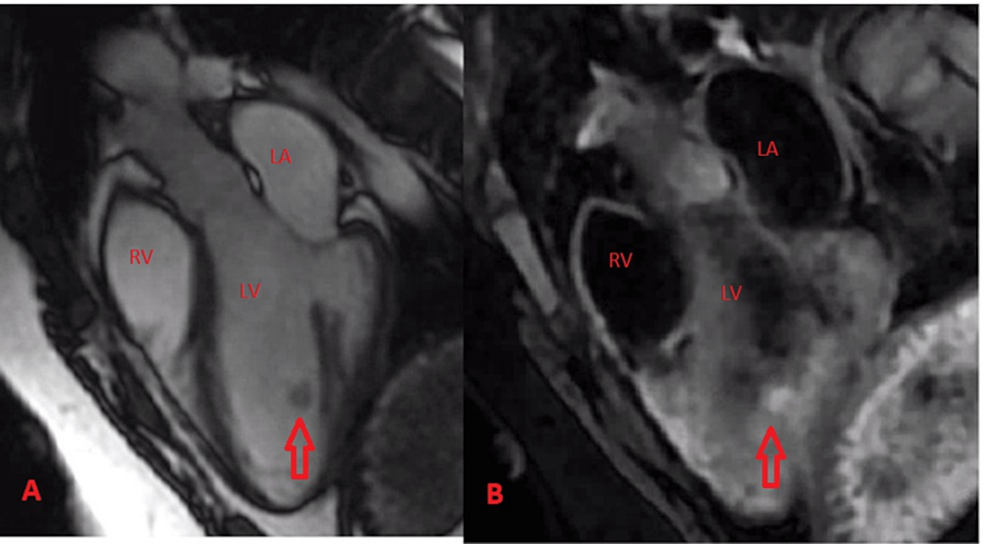
Cardiac MRI imaging A. Steady-state free precession (SSFP) imaging in a 3-chamber orientation demonstrates the mass to be isointense to the myocardium. B. Fat-suppressed black blood T2 weighted imaging (DIR spin echo) demonstrating the mass to by hyperintense compared to the myocardium. LV: Left ventricle, LA: Left atrium, RV: Right Ventricle, DIR: Double inversion recovery

A cardiac MRI revealed a spherical ~8 × 10 × 8 mm hypermobile mass located in the mid-inferior to apical-inferior portion of the cavity of the left ventricle. The mass projected ~13 mm into the body of the left ventricle. The mass appeared to be attached to an incompletely visualized thin stalk with characteristics of a myxoma (Figures 1, 2).

The patient underwent open heart surgery for excision of the mass. There were no post-operative complications. On gross examination, the mass measured approximately 1 cm, was fleshy and had an element of thrombus.

**Figure 3:**
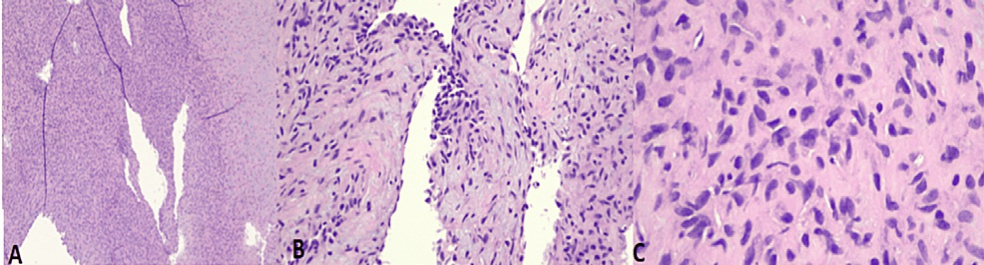
Pathology slides on Hematoxylin and Eosin staining Magnification: A. x4 , B. x20 , C x40. A well-circumscribed nodule composed of bland spindle cells and fibro myxoid stroma. A few dilated luminal spaces with papillary fronds lined by flat endothelial cells are present.

**Figure 4:**
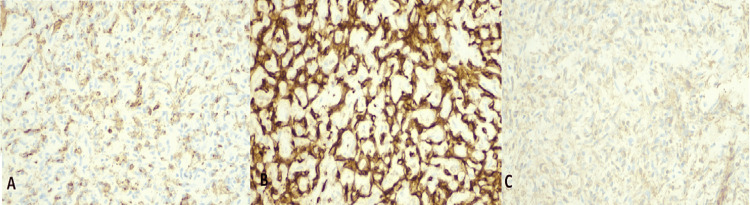
Immunohistochemistry study results (x20 magnification) A.CD34+ , B. CD31+, C. Smooth muscle actin

The pathology report described an organizing thrombus with papillary endothelial hyperplasia (Masson’s tumor) (Figure 3) with immunohistochemistry positive for CD34. Luminal lining cells were positive for ERG, CD31, and smooth muscle actin (Figure 4). The patient was discharged on aspirin, metoprolol, and colchicine with a follow-up with the cardiology and cardiothoracic surgery departments. She had no recurrent symptoms on follow-up.

## Discussion

There are few case reports of IPEH in the heart. Previous cases have been reported in the left atrium (LA) [4-6]. There’s only one reported case in the left ventricle arising from a hemangioma [2]. The rationale for the LA being a potential site is because of the trabecular lining of the left atrial appendage that predisposes to blood stasis and thrombi formation, thus leading to possible IPEH [4]. In our case, there were no predisposing factors for thrombi formation and the patient had normal ventricular function.

Cardiac masses can be challenging to diagnose. Biopsies are invasive so we must rely on imaging to characterize a lesion. Among different imaging modalities, Cardiac magnetic resonance (CMR) is ideal for differentiating soft tissue. There are a few cases describing the appearance of IPEH on CMR. If the tumor arises from a thrombus or hemangioma, you could expect imaging to show characteristics of these. Features of a thrombus typically include no uptake on late Gadolinium enhancement (LGE) and low or high intensity in T1 and T2- weighted imaging [7]. In this case, pathology showed IPEH within an organizing thrombus, but CMR showed characteristics of a myxoma. Few cases have described IPEH's appearance on CMR. Oza et al. described a pure IPEH in the right ventricle as being isointense compared to the myocardium on double inversion recovery (DIR), slightly hyperintense on SSFP, and homogenous LGE [8]. This description varies from what we found. More reports of IPEH appearance on CMR are needed. Echocardiography also plays a role as it can help in assessing intracavitary flow and when contrast is added, it can differentiate a tumor from a thrombus since the latter has low vascularity [9].

Although IPEH is considered a benign tumor, management of left-sided tumors is generally surgical resection due to the risk of embolization [10]. Other factors to consider are obstructive symptoms such as syncope, congestive heart failure, and pulmonary edema secondary to obstruction of the mitral valve.

## Conclusions

Cardiac Masson’s tumor is exceedingly rare with only a few cases described in the left ventricle. Despite advances in imaging, excisional biopsy is still the diagnostic gold standard. This case posed a challenge for imaging interpretation because it was reported as a potential myxoma with observed thrombi characteristics. Although it is a benign tumor, excision is recommended due to the risk of embolization.
